# NEWS AND NOTICES

**Published:** 2011-09

## News

### Photo and video competition winners

**Figure:**
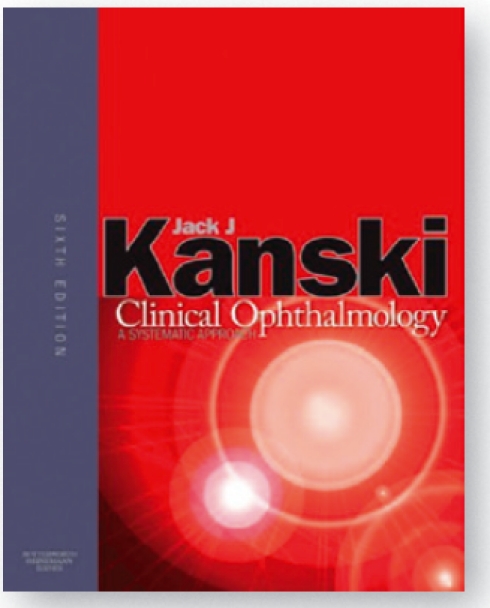


Thank you very much to all our readers who entered this competition. The winner of the video competition is Stephen Thompson of the Universidade Lúrio in Mozambique, who will receive a copy of Kanski's Clinical Ophthalmology kindly donated by Elsevier (worth UK £164). First prize in the photo competition goes to Andrew Potter, Benin. Tatowela Mmoloki, Botswana, Mohsin Alam, India and Sr MG King, South Africa share second place. All winners will receive book prizes.

### Have your say: low vision

Our March 2012 issue is about managing adults and children with low vision. Have you had a useful or interesting experience in low vision that you would like to share with other readers? Do you have any questions you would like to ask our experts? Write to: The Editor, International Centre for Eye Health, London School of Hygiene and Tropical Medicine, London WC1E 7HT, UK. Email: **editor@cehjournal.org** Deadline: 15 January 2012.

### Get your own copy

Do you get your own copy of the *Community Eye Health Journal?* Do you know anyone else who would like their own, free copy? Or have you moved or changed jobs? Send your up-to-date details to Anita Shah, International Centre for Eye Health, London School of Hygiene and Tropical Medicine, London WC1E 7HT, UK. Email: admin@cehjournal.org

### Online Sightsavers journal

*Insight Plus* is a practitioner journal consisting of learning and best practice from across the many different Sightsavers programmes in Africa, Asia, and the Caribbean. Each issue focuses on a different theme and is published twice a year. The journal includes case studies, opinion papers, and learning summaries. Please information, please contact: learning@sightsavers.org for information. Issues may be downloaded from www.sightsavers.org/InsightPlus

## Courses

### Community Eye Health Institute, University of Cape Town, South Africa

For information about VISION 2020 certificate courses in 2012, a postgraduate diploma in community eye health (PGDip) in 2013, or a Masters in Public Health (community eye health) in 2013, contact Zanele Magwa, Community Eye Health Institute, University of Cape Town, Private Bag 3, Rondebosch 7700, South Africa. Tel: +27 21 404 7735. Email: ntombizanele.magwa@uct.ac.za

### International Centre for Eye Health

**MSc in Public Health for Eye Care**.

From September 2012 to September 2013 or part-time over two years. Apply before April 2012. For scholarships and details of application, write to: Registry, LSTHM, Keppel Street, London WC1E 7HT, UK. Tel: +44 207 299 4646 or visit www.lshtm.ac.uk/prospectus/masters/mscphec.html

### Kilimanjaro Centre for Community Ophthalmology (KCCO), Tanzania

For information on courses, contact Genes Mng'anya, KCCO, Good Samaritan Foundation, PO Box 2254 Moshi, Tanzania. Tel: +255 27 275 3547. Email: genes@kcco.net or visit www.kcco.net

### Lions SightFirst Eye Hospital, Nairobi, Kenya

**Small incision cataract surgery for ophthalmologists wishing to upgrade from ECCE**. Duration: 1 month. Courses run every month. Cost: US $1,000 for tuition and US$ 500-700 for accommodation and meals. Write to: The Training Coordinator, Lions Medical Training Centre, Lions SightFirst Eye Hospital, PO Box 66576-00800, Nairobi, Kenya, call +254 20 418 32 39, or email training@lionsloresho.org

### International Centre for Advancement of Rural Eye Care, LV Prasad Eye Institute, India

Community Eye Health Diploma (6 months, US $8,000) and Masters (11 months, US $21,000). Courses start in January 2012. Contact S Sheeladevi, International Centre for Advancement of Rural Eye Care, LV Prasad Eye Institute, Kismatpur Campus, LV Prasad Marg, Hyderabad 500 034, India. Email: sheela@lvpei.org

### Lions Aravind Institute of Community Ophthalmology

**Instrument maintenance courses** with a trainee: trainer ratio of 1:1. Courses start on 1 Feb, 1 Apr, 1 June, 1 Aug, 1 Oct and 1 Dec 2012. Duration: Four weeks. Cost: US $400 (including tools). Visit www.aravind.org/education/coursedetails.asp or write to: Prof V Srinivasan, LAICO, 72, Kuruvikaran Salai, Gandhi Nagar, Madurai 625 020, Tamil Nadu, India. Email: v.srinivasan@aravind.org

